# Understanding the Role of Propulsion in the Prediction of Front-Crawl Swimming Velocity and in the Relationship Between Stroke Frequency and Stroke Length

**DOI:** 10.3389/fphys.2022.876838

**Published:** 2022-04-27

**Authors:** Jorge E. Morais, Tiago M. Barbosa, Alan M. Nevill, Stephen Cobley, Daniel A. Marinho

**Affiliations:** ^1^ Department of Sport Sciences, Instituto Politécnico de Bragança, Bragança, Portugal; ^2^ Research Center in Sports Health and Human Development (CIDESD), University of Beira Interior, Covilhã, Portugal; ^3^ Faculty of Education, Health, and Wellbeing, University of Wolverhampton, Wolverhampton, United Kingdom; ^4^ Discipline of Exercise and Sport Science, Faculty of Health Sciences, The University of Sydney, Sydney, NSW, Australia; ^5^ Department of Sport Sciences, University of Beira Interior, Covilhã, Portugal

**Keywords:** youth, swimming, technique, performance, stroke parameters

## Abstract

**Introduction:** This study aimed to: 1) determine swimming velocity based on a set of anthropometric, kinematic, and kinetic variables, and; 2) understand the stroke frequency (SF)–stroke length (SL) combinations associated with swimming velocity and propulsion in young sprint swimmers.

**Methods:** 38 swimmers (22 males: 15.92 ± 0.75 years; 16 females: 14.99 ± 1.06 years) participated and underwent anthropometric, kinematic, and kinetic variables assessment. Exploratory associations between SL and SF on swimming velocity were explored using two two-way ANOVA (independent for males and females). Swimming velocity was determined using multilevel modeling.

**Results:** The prediction of swimming velocity revealed a significant sex effect. Height, underwater stroke time, and mean propulsion of the dominant limb were predictors of swimming velocity. For both sexes, swimming velocity suggested that SL presented a significant variation (males: F = 8.20, *p* < 0.001, η^2^ = 0.40; females: F = 18.23, *p* < 0.001, η^2^ = 0.39), as well as SF (males: F = 38.20, *p* < 0.001, η^2^ = 0.47; females: F = 83.04, *p* < 0.001, η^2^ = 0.51). The interaction between SL and SF was significant for females (F = 8.00, *p* = 0.001, η^2^ = 0.05), but not for males (F = 1.60, *p* = 0.172, η^2^ = 0.04). The optimal SF–SL combination suggested a SF of 0.80 Hz and a SL of 2.20 m (swimming velocity: 1.75 m s^−1^), and a SF of 0.80 Hz and a SL of 1.90 m (swimming velocity: 1.56 m s^−1^) for males and females, respectively. The propulsion in both sexes showed the same trend in SL, but not in SF (i.e., non-significant variation). Also, a non-significant interaction between SL and SF was observed (males: F = 0.77, *p* = 0.601, η^2^ = 0.05; females: F = 1.48, *p* = 0.242, η^2^ = 0.05).

**Conclusion:** Swimming velocity was predicted by an interaction of anthropometrics, kinematics, and kinetics. Faster velocities in young sprinters of both sexes were achieved by an optimal combination of SF–SL. The same trend was shown by the propulsion data. The highest propulsion was not necessarily associated with higher velocity achievement.

## Introduction

Competitive swimming is a time-based sport where the athlete must travel a given distance at maximum velocity (Seifert et al., 2007). Power input and transport energy cost are the two main underlying factors that allow faster velocities to be achieved:

$$v=\frac{{\dot{E}}_{tot}}{C}$$ (1)

in which v is the swimming velocity (in m·s^−1^), ${\dot{\text{E}}}_{\text{tot}}$ is the energy expenditure (in ml·kg^−1^·m^−1^; also known as total power input–W), and C is the energy cost of swimming (in J·kg^−1^·m^−1^) (Barbosa et al., 2010). Thus, swimming performance has a strong relationship with the mean swimming velocity across a given stroke event (Craig et al., 1985).

Predicting swimming velocity is the main goal of researchers and coaches (Morais et al., 2012; Abbes et al., 2018). Swimming velocity is highly dependent from anthropometric variables (Nevill et al., 2020; Oliveira et al., 2021), kinematics and motor control (Morais et al., 2012; Figueiredo et al., 2016; Silva et al., 2019), energetics/efficiency (Figueiredo et al., 2016; Barbosa et al., 2019), and dry-land strength and power (Girold et al., 2012; Strzala et al., 2019). However, most recent research trends highlighted swimming performance as a holistic phenomenon that is strongly dependent from the interaction between several variables of different scientific fields (Figueiredo et al., 2016; Morais et al., 2021). The potential interaction between swimming performance determinants (i.e., scientific fields, domains, and variables that may determine or predict swimming performance) provides the platform for different and multiple patterns of behavior to emerge on an individual basis. There is an interplay among several variables that ultimately will affect the swimming velocity (Morais et al., 2012; Figueiredo et al., 2016). Therefore, small gains by each variable can trigger a change in the interplay among the components of the system which will ultimately “affect” the variable being determine (in this case swimming velocity). It has been indicated, both experimentally (Tsunokawa et al., 2019; Morais et al., 2020a) and numerically (Bilinauskaite et al., 2013; Cohen et al., 2018), that a greater propulsion is related to faster swimming velocity. A study conducted by Santos et al. (2021) reviewed the state of the art about human propulsion in competitive swimming. Propulsion in swimming refers to the force generated by the swimmer through the actions of upper and lower limbs to promote forward motion (Barbosa et al., 2020). However, there is little awareness of the role that propulsion can play when it interacts with other variables. Thus, being propulsion a key-factor for the swimming velocity improvement, it seems of major importance understanding the magnitude of its influence when interacted with other key-factors.

As with any other cyclic phenomena, the mean swimming velocity depends on the frequency and length of the stroke:

$$\bar{v}=SF\cdot SL$$ (2)

In which $\bar{\text{v}}$ is the mean swimming velocity (in m·s^−1^), SF is the stroke frequency (in Hz), and SL is the stroke length (in m) (Craig and Pendergast, 1979). Therefore, the mean swimming velocity can be improved by increasing SF, SL, or both concurrently. In freestyle events (i.e., front-crawl), it is known that increasing swimming velocity, based on higher SF, leads to a higher energy cost of transportation (Wakayoshi et al., 1993; Komar et al., 2012). Conversely, increasing swimming velocity by increasing SL is associated with a small increment in energy cost (Barbosa et al., 2008). Thus, understanding the relationship between SF and SL is of paramount importance to reach a certain mean swimming velocity (Craig and Pendergast, 1979; Dormehl and Osborough, 2015).

Overall, in all swimming events (i.e., strokes and distances), swimmers can use two main pacing strategies: 1) higher SF and shorter SL, or 2) lower SF and longer SL (Maglischo, 2003; Hellard et al., 2008). Swimmers can even trade-off SF and SL during an event. Whenever an increase in SF is observed, there is often a consequential tendency for SL to decrease (Seifert et al., 2010). Contrastingly, if SF decreases, SL tends to increase (Psycharakis et al., 2008). This happens because swimmers take more time to complete the full stroke cycle (Alberty et al., 2011). In the specific case of freestyle sprinters racing the 50 m event, it was shown that elite swimmers (participating in major competitions such as European and World championships) present an all-out strategy (Simbaña-Escobar et al., 2018; Morais et al., 2022). That is, swimmers exhibit a positive pacing–swimming velocity decrease over time, with a SF decrease and a SL increase over time (Morais et al., 2022). Nonetheless, it was suggested that swimmers may needed to change the SF–SL combination to maintain a given pace (Dekerle et al., 2005). Moreover, whenever swimmers fail to maintain SF, they are often advised to maintain SL to minimize the decrease in swim velocity (Seifert et al., 2005).

Besides the spatial-temporal factors mentioned above, one can argue that other factors can account for dynamics in the SF–SL relationship. For instance, upper limb propulsion may also play an influential role. Overall, it was experimentally shown that propulsion presents a significant and positive relationship with swim velocity (Morais et al., 2020a; Koga et al., 2020). That is, higher propulsion by the upper limbs leads to faster swim velocities. Notwithstanding, it must be pointed out that propulsion generated by the upper limbs account for 90% of the swim velocity (lower limbs actions are responsible for remaining 10%) (Deschodt et al., 1999). Regarding the influence that propulsion may have on SF and SL, it was suggested that the capability to keep a given propulsion intensity throughout the in-water phase of the stroke cycle (i.e., pull and push motion–propulsion) can explain reductions in SF, subsequently leading to a longer SL. Alternatively, a shorter SL can be associated with a lower capability to generate sufficient propulsion necessary to overcome drag (Craig et al., 1985). Others verified that after 4 years of high-velocity training, the participants of their study were able to swim at a given submaximal velocity with a slower SF (i.e., with a longer SL) (Termin and Pendergast, 2000). One can speculate that at least one key-factor for this was the increase of propulsion generated by the swimmers that allowed to swim at faster velocities with slower SF. However, beside such assumptions, one cannot find in the literature evidence about the role that propulsion plays in this SF–SL relationship. As a result, it is logical that understanding the role that propulsion may have on the SF–SL relationship is of paramount importance for swimmers and coaches to support practitioners in designing and developing training programs. This will allow to identify SF–SL combinations that might elicit better performances.

The purposes of the present study were to (1): predict swimming velocity based on a set of anthropometric, kinematic, and kinetic variables, and; 2) understand the SF–SL combinations associated with swimming velocity and propulsion in young sprint swimmers. It was hypothesized that: 1) kinetic variables would be retained as swimming velocity predictors, interacted with anthropometrics and kinematics (all of them with a positive and significant effect), and; 2) the fastest swims are characterized by the highest SF but not the lowest SL, and propulsion plays a determinant and positive role in the SF–SL ratio and swimming velocity.

## Methods

### Participants

The participants were 38 swimmers (22 males: 15.92 ± 0.75 years-old, FINA points: 566.77 ± 56.82 in the 100 m freestyle event–short course meter swimming pool; 16 females: 14.99 ± 1.06 years-old, FINA points: 602.25 ± 77.35 in the 100 m freestyle event–short course meter swimming pool). Swimmers were recruited from a national squad that competed at international championships and contained age-group national champions and record holders, i.e., Tier 3 (McKay et al., 2022). The inclusion criteria for the participants were: 1) being male and female sprint specialists in their age-group in freestyle sprinting events, and; 2) having participated in daily training sessions from the beginning of the season and without injuries. Participants had more than 5 years of competitive experience; trained six to seven swimming sessions per week; and, had at least one dry-land strength and conditioning session per week. Swimmers were informed about the study procedures as well as the possible risks that could arise from the study. Parents or guardians as well as the swimmers themselves provided informed consent. All procedures were in accordance with the Declaration of Helsinki regarding human research, and the University Ethics Board approved the research design.

### Experimental Design

This was a cross-sectional study. After a standardized 1,000 m warm-up, swimmers completed three all-out trials of 25 m freestyle with a push-off start, the fastest trial being used for analysis. Swimmers were instructed to hold their breath during such intermediate distance to avoid modifications in coordination due to breathing. Rest time between trials was 30 min. In-water warm-up and trial performance took place in a 25 m indoor swimming pool (water temperature: 27.5°C; air temperature: 26.0°C; relative humidity: 67% prior to the swimming performance assessment). As part of the trial performance, kinematic and kinetic variables were measured.

### Anthropometric Assessment

Participants initially underwent an anthropometric assessment. At this time, the swimmers’ hand dominancy was assessed by self-report as suggested elsewhere (Morais et al., 2020b). Height (H, in cm) was measured as the distance between the vertex to the floor (with the swimmers in the orthostatic position) using a digital stadiometer (SECA, 242, Hamburg, Germany). Body mass (BM, in kg) was measured on a digital scale (TANITA, BC-730, Amsterdam, Netherlands). The swimmer’s arm span (AS, in cm) was measured using digital photogrammetry. Swimmers were placed in an orthostatic position, with both arms in lateral abduction at a 90° angle with the trunk. Both arms and fingers were fully extended. The distance between the tip of each third finger was measured with a dedicated software (Udruler, AVPSoft, United States) (Morais et al., 2020b). For hand surface area (HSA, in cm^2^), swimmers placed their hands onto a copy machine for surface area scanning. Each HSA was determined using the digital scan by a dedicated software (Udruler, AVPSoft, United States) (Morais et al., 2012).

### Stroke Kinematic Assessment

To determine maximum velocity, a speedometer apparatus (Swim speedo-meter, Swimsportec, Hildesheim, Germany) was attached to the swimmers’ hip (Barbosa et al., 2019). In-house built software (LabVIEW^®^, v. 2010), previously acquired (*f* = 50 Hz), displayed velocity-time data across each swimmer’s trial (Barbosa et al., 2011). Data was exported to an interface by a 12-bit resolution acquisition card (USB-6008, National Instruments, Austin, Texas, United States). Afterwards, data was imported into signal processing software (AcqKnowledge v. 3.9.0, Biopac Systems, Santa Barbara, United States). Signals were handled using a Butterworth fourth order low-pass filter (cut-off: 5Hz, based on the analysis of the residual error vs cut-off frequency output) (Barbosa et al., 2019). A video camera (Sony FDR-X3,000, Japan) was also attached to a rail at the edge of the swimming pool and recorded swimmers in the sagittal plane. The camera was synchronized with the velocity-time software by a light signal. When the speedo-meter starts acquiring data, a light is enhanced in the software. The camera filmed this moment so that afterwards the velocity-time curve can be synchronized with the video.

The following stroke kinematic variables were determined via assessment of three consecutive stroke cycles during the intermediate 15 m of the swimming pool. The in-water phase of the stroke cycle was considered to start at the hand’s entry and finishes at the hand’s exit. Swimming velocity (v, in m·s^−1^) was retrieved from the velocity-time curve. Based on video recording assessment, stroke frequency (SF, in Hz) was calculated by the number of cycles per unit of time, specifically the time required to complete a full cycle (f = 1/P; where P is the period), later converted to Hz. The stroke length (SL, in m) was calculated as SL = v/SF (Craig and Pendergast, 1979). The intra-cyclic variation of the horizontal swimming velocity (dv, in %) was computed as the coefficient of variation (CV): CV = one standard deviation/mean * 100 (Barbosa et al., 2010). The underwater stroke time of each upper limb (UST_dominant_ and UST_non-dominant_, in s) was computed as the time spent between the entry of the hand in the water and its exit. Then, the mean of both upper limbs was calculated (UST_stroke cycle_, in s).

### Propulsion Assessment

Kinetic data were acquired simultaneously with kinematic data. Thus, the same three consecutive stroke cycles were analyzed. Pressure sensors (Swimming Technology Research, United States; https://swimmingtechnology.com/aquanexanalysis/) were used to measure propulsion (*f* = 100 Hz) (Havriluk, 2013). This system is based on sensors that estimate in-water pressure (Havriluk, 1988; Barbosa et al., 2020). The sensors were placed between the third and fourth metacarpals to measure the pressure differential between the palmar and dorsal surfaces. This location is assumed as being a good proxy for the application point of propulsion vector on the hand (Gourgoulis et al., 2013). The application of additional sensors on each hand was avoided as it can affect technique, due to cabling surrounding the upper limb. Additional sensors may change the geometry and volume of the hand, impacting the ecological validity of the propulsion data. At the beginning of each performance trial, swimmers were asked to keep their hands immersed at a depth of 0.50 m for 10 s to calibrate the system. The pressure sensor data were transferred to the Aquanex software (Aquanex v. 4.2 C1211, Richmond, United States) by an A/D converter (Morais et al., 2020b). Afterwards, time-force series were imported into a signal processing software (AcqKnowledge v. 3.9.0, Biopac Systems, Santa Barbara, United States). Signals were again handled using a Butterworth fourth order low-pass filter (cut-off: 5 Hz). For each dominant and non-dominant in-water phase of the stroke cycle, the mean propulsion (F_mean_dominant_ and F_mean_non-dominant_, in N) and peak force (F_peak_dominant_ and F_peak_non-dominant_, in N) were determined. Afterwards, the F_mean_stroke cycle_ (the mean force produced in one full stroke cycle, in N) was calculated. The intra-cyclic variation of the propulsion of each upper limb (dF_dominant_ and dF_non-dominant_, in %) was computed based on equation 3. Then, the mean across both upper limbs was calculated (dF_mean_stroke cycle_, in %).

### Statistical Analysis

Initially, the Kolmogorov-Smirnov and the Levene tests were used to assess normality and homoscedasticity, respectively. Descriptive statistics means and one standard deviation (±1SD) were calculated. Exploratory associations between SL and SF on swimming velocity were explored using the two-way ANOVA (independent for males and females) (*p* < 0.05). Swimming velocity was entered as the dependent variable with both SL and SF categorized (rounded) as independent variables (males: 13 categories for SL and 4 for SF; females: 8 categories for SL and 3 for SF). “Rounding” consists of converting continuous variables (in this case SF and SL) into categories. Later, a similar analysis was performed on the propulsion to verify which values corresponded to a given swimming velocity rounded by SL and SF. For both swimming velocity and propulsion non-estimable means were not considered. Thus, only the combinations observed for both males and females were analyzed. Eta square (η^2^) was used as an effect size index and interpreted as: 1) without effect if 0 < η^2^ ≤ 0.04; 2) minimum if 0.04 < η^2^ ≤ 0.25; 3) moderate if 0.25 < η^2^ ≤ 0.64 and; 4) strong if η^2^ > 0.64 (Ferguson, 2009).

To calculate the swimming velocity, multilevel modeling was used. Swimming velocity was defined as the dependent variable. The remaining anthropometric, kinematic (except SF and SL), and kinetic variables were defined as independent or predictor variables (*p* < 0.05). The analysis was performed using the MLwiN multilevel modeling software (Bristol, United Kingdom). Multilevel modeling is an extension of ordinary multiple regression in which data have a hierarchical or clustered structure. The hierarchy consists of units or measurements grouped at different levels. In the current study, it is assumed that the swimmers are a random sample, representing the level 2 units, and the swimmers’ repeated measurements (three consecutive stroke cycles), the level 1 units (Morais et al., 2020a). The 95% confidence intervals (95CI) were computed. A multicollinearity phenomenon was not detected since the independent variables were all computed independently from the dependent one. Differential calculus was used to estimate the point at which the dependent variables peaked when a significant quadratic association was identified (i.e., H and F_mean_dominant_) (Alcock, 2016).

## Results

Table 1 presents the descriptive statistics (mean ± 1SD) for all measured variables. Males presented higher anthropometrics and larger kinematics and kinetics than their female counterparts (Table 1).

**TABLE 1:** Descriptive data for all the variables assessed by sex. In-water variables include the data from the three stroke cycles measured.

	Males			Females		
**Anthropometrics**	**Mean±1SD**			**Mean±1SD**		
BM [kg]	68.93 ± 6.99			56.66 ± 5.94		
H [cm]	176.91 ± 5.57			162.63 ± 6.80		
AS [cm]	182.81 ± 8.24			167.56 ± 6.96		
HSA_dominant_ [cm^2^]	139.04 ± 10.07			114.52 ± 11.84		
HSA_non-dominant_ [cm^2^]	140.57 ± 11.72			114.05 ± 11.94		
	**Mean±1SD**			**Mean±1SD**		
**Kinematics**	**1st stroke cycle**	**2nd stroke cycle**	**3rd stroke cycle**	**1st stroke cycle**	**2nd stroke cycle**	**3rd stroke cycle**
v [m·s^−1^]	1.63 ± 0.07	1.63 ± 0.10	1.62 ± 0.08	1.43 ± 0.08	1.43 ± 0.08	1.41 ± 0.08
dv [%]	10.26 ± 4.59	10.06 ± 4.71	9.73 ± 4.35	7.99 ± 1.92	8.10 ± 2.27	8.72 ± 1.80
SF [Hz]	0.86 ± 0.06	0.86 ± 0.08	0.86 ± 0.08	0.81 ± 0.05	0.81 ± 0.05	0.82 ± 0.04
SL [m]	1.91 ± 0.14	1.91 ± 0.13	1.89 ± 0.16	1.76 ± 0.10	1.77 ± 0.09	1.73 ± 0.09
UST_dominant_ [s]	0.84 ± 0.11	0.85 ± 0.10	0.86 ± 0.10	0.77 ± 0.11	0.77 ± 0.11	0.79 ± 0.10
UST_non-dominant_ [s]	0.80 ± 0.08	0.81 ± 0.09	0.82 ± 0.07	0.74 ± 0.08	0.75 ± 0.09	0.75 ± 0.11
UST_stroke cycle_ [s]	0.82 ± 0.09	0.83 ± 0.09	0.84 ± 0.08	0.76 ± 0.09	0.76 ± 0.10	0.77 ± 0.10
		**Mean±1SD**			**Mean±1SD**	
**Propulsion**	**1st stroke cycle**	**2nd stroke cycle**	**3rd stroke cycle**	**1st stroke cycle**	**2nd stroke cycle**	**3rd stroke cycle**
F_mean_dominant_ [N]	40.26 ± 6.34	38.88 ± 6.34	39.22 ± 7.57	33.64 ± 4.31	32.70 ± 4.45	32.86 ± 4.36
F_peak_dominant_ [N]	65.77 ± 10.69	62.73 ± 9.87	63.89 ± 11.21	57.13 ± 8.37	54.16 ± 8.14	54.09 ± 8.25
dF_dominant_ [%]	43.69 ± 10.76	44.18 ± 9.19	45.53 ± 10.77	43.94 ± 8.23	43.98 ± 9.05	43.18 ± 8.60
F_mean_non-dominant_ [N]	37.35 ± 6.42	37.45 ± 5.57	37.04 ± 6.02	32.67 ± 5.05	33.59 ± 5.24	31.93 ± 4.41
F_peak_non-dominant_ [N]	65.60 ± 11.67	64.27 ± 8.90	63.07 ± 9.29	55.42 ± 10.37	54.68 ± 10.78	53.62 ± 9.79
dF_non-dominant_ [%]	53.62 ± 11.80	49.18 ± 8.30	50.08 ± 9.20	46.91 ± 11.27	43.48 ± 10.31	45.57 ± 9.46
F_mean_stroke cycle_ [N]	38.80 ± 5.50	38.16 ± 4.85	38.23 ± 5.70	33.16 ± 3.98	33.15 ± 4.20	32.09 ± 2.97
dF_mean_stroke cycle_ [%]	48.65 ± 9.86	46.68 ± 6.88	47.68 ± 6.48	45.42 ± 8.59	43.73 ± 8.22	44.37 ± 7.99

BM–body mass; H–height; AS–arm span; HSA_dominant_–hand surface area of the dominant limb; HSA_non-dominant_–hand surface area of the non-dominant limb; v–swim velocity; dv–intra-cyclic variation of the swim velocity; SF–stroke frequency; SL–stroke length; UST_dominant_–underwater stroke time of the dominant limb; UST_non-dominant_–underwater stroke time of the non-dominant limb; UST_stroke cycle_–mean underwater stroke time of the stroke cycle; F_mean_dominant_–mean propulsion of the dominant upper-limb; F_peak_dominant_–peak propulsion of the dominant upper-limb; dF_dominant_–intra-cyclic variation of the dominant upper-limb force; F_mean_non-dominant_–mean propulsion of the non-dominant upper-limb; F_peak_non-dominant_–peak propulsion of the non-dominant upper-limb; dF_non-dominant_–intra-cyclic variation of the non-dominant upper-limb force; F_mean_stroke cycle_–mean propulsion of the full stroke cycle; dF_mean_stroke cycle_–mean intra-cyclic variation of the full stroke cycle force.

The results of the multilevel regression analysis that predict swimming velocity are reported in Table 2. A significant sex effect was verified (estimate = 0.2003, 95CI: 0.1309 to 0.2697, *p* < 0.001) (Table 2). The UST_dominant_ was the independent variable that presented the highest effect (estimate = -0.1787, 95CI: 0.3504 to -0.0070, *p* = 0.0207). Differential calculus showed that the optimal value for the F_mean_dominant_ was 34.75 N and for the H was 174.67 cm. From those values onwards the swimming velocity decreased.

**TABLE 2:** Fixed effects of the final swimming velocity model computed with standard errors (SE), 95% confidence intervals (95CI), test-score (z-score), and significance value (p).

	Estimate	SE	z-score	P	95CI
Sex	0.2003	0.0354	5.7	<0.001	0.1309 to 0.2697
H	0.1048	0.0357	2.9	<0.001	0.0348 to 0.1747
H^2^	-0.0003	0.0001	-3.0	0.0017	-0.0005 to -0.0001
UST_dominant_	-0.1787	0.0876	-2.0	0.0207	-0.3504 to -0.0070
F_mean_dominant_	0.0139	0.0064	2.2	0.0149	0.0014 to 0.0264
F_mean_dominant_^2^	-0.0002	0.0001	-2.0	0.0228	-0.0004 to -0.000004

H–height; UST_dominant_–underwater stroke time (dominant upper-limb); F_mean_dominant_–mean propulsion produced by the dominant upper-limb.

Table 3 shows the swimming velocity and propulsion categorization by SL round, SF round, and its interaction. Regarding swimming velocity for both sexes, SL (males: F = 8.20, *p* < 0.001, η^2^ = 0.40; females: F = 18.23, *p* < 0.001, η^2^ = 0.39) and SF (males: F = 38.20, *p* < 0.001, η^2^ = 0.47; females: F = 83.04, *p* < 0.001, η^2^ = 0.51) presented significant effects. The interaction between SL and SF was significant for females (F = 8.00, *p* = 0.001, η^2^ = 0.05), but not for males (F = 1.60, *p* = 0.172, η^2^ = 0.04). Regarding the propulsion for both sexes, the same trend was verified in SL (males: F = 2.54, *p* = 0.013, η^2^ = 0.32; females: F = 3.07, *p* < 0.012, η^2^ = 0.34), but not in SF (males: F = 1.91, *p* = 0.144, η^2^ = 0.06; females: F = 0.78, *p* = 0.466, η^2^ = 0.02). The interaction between SL and SF was non-significant for both sexes (males: F = 0.77, *p* = 0.601, η^2^ = 0.05; females: F = 1.48, *p* = 0.242, η^2^ = 0.05) (Table 3).

**TABLE 3:** Male and female two-way ANOVAs considering swimming velocity and propulsion by SL round, SF round, and their interaction (see Figure 1).

	Males			Females		
Swim velocity	**F-ratio**	**p**	**η** ^**2**^	**F-ratio**	**p**	**η** ^**2**^
SL round	8.20	<0.001	0.40	18.23	<0.001	0.39
SF round	38.20	<0.001	0.47	83.04	<0.001	0.51
SL * SF round	1.60	0.172	0.04	8.40	0.001	0.05
*R* ^2^	0.831			0.889		
Mean square error	0.002			0.001		
Propulsion						
SL round	2.54	0.013	0.32	3.07	0.012	0.34
SF round	1.91	0.144	0.06	0.78	0.466	0.02
SL * SF round	0.77	0.601	0.05	1.48	0.242	0.05
*R* ^2^	0.576			0.427		
Mean square error	18.733			10.327		

Propulsion–correspond to the F_mean_stroke cycle_ (mean propulsion of the full stroke cycle); SL, stroke length; SF, stroke frequency; η^2^–eta square (effect size index).

**FIGURE 1:**
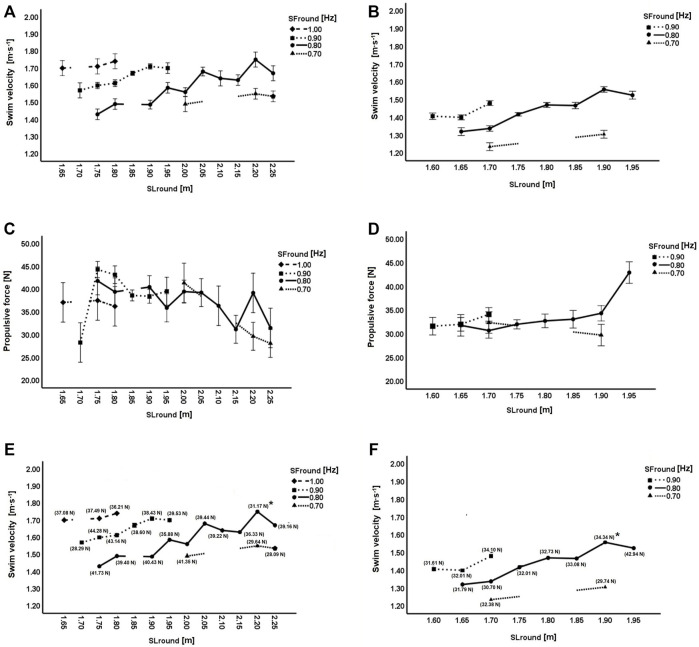
Swimming velocity rounded by SF and SL: Panel **(A)** males; Panel **(B)** females. Propulsion rounded by SF and SL: Panel **(C)** males; Panel **(D)** females. Swim velocity rounded by SF and SL, with correspondent propulsion (F_mean_stroke cycle_) in each SL–SF combination: Panel **(E)** males; Panel **(F)** females. * - Panel **(E)**: highest swimming velocity achieved by male swimmers (1.75 m s^−1^); Panel **(F)**: highest swimming velocity achieved by female swimmers (1.56 m s^−1^). Error bars represent one standard deviation.

Figure 1 depicts the swimming velocity categorized by SL and SF (Panel A–males; Panel B–females). In males (Figure 1–panel A) the SF–SL combination corresponding to the fastest swimming velocity suggested a SF of 0.80 Hz and a SL of 2.20 m (swimming velocity: 1.75 m s^−1^). For females (Figure 1–panel B), the SF–SL combination indicated a SF of 0.80 Hz and a SL of 1.90 m (swimming velocity: 1.56 m s^−1^). Figure 1 also depicts the propulsion categorized by SL and SF (Panel C–males; Panel D–females). The highest propulsion in males (44.28 N) was observed with a combination of 0.90 Hz (SF) and 1.75 m (SL), and in females (42.94 N) by combining a SF of 0.80 Hz with a SL of 1.95 m. Considering the swimming velocity against SL, and taking into account the propulsion delivered at each SF (Panel E–males; Panel F–females), it is possible to observe that the SF–SL combination to achieve the fastest velocity was not the one providing the highest propulsion (Figure 1).

## Discussion

The aim of this study was to predict swimming velocity based on a set of anthropometric, kinematic, and kinetic variables, and understand the SF–SL combinations associated with swimming velocity and propulsion in young sprint swimmers. The main findings are that swimming velocity model produced a significant sex effect (being males fastest than females) and retained as predictors the anthropometrics (H), kinematics (UST_dominant_), and kinetics (F_mean_dominant_). The fastest swimming velocity for both sexes was not achieved either at the highest SF or SL. Moreover, the highest propulsion was not responsible for delivering the fastest swimming velocity.

Swimming velocity retained as significant predictors sex, H, H^2^, UST_dominant_, F_mean_dominant_, and F_mean_dominant_^2^. Since the sample included post-pubertal swimmers (males: 15.92 ± 0.75 years-old; females: 14.99 ± 1.06 years-old) a sex effect was expected. Thus, unsurprisingly, males were faster than females. Height was the best predictor retained by the model. The literature demonstrates that the fastest swimmers are taller and have a wider arm span and larger body dimensions in relation to the upper body (Nevill et al., 2015; Figueiredo et al., 2016). The UST_dominant_ and the F_mean_dominant_ (kinematics and kinetics, respectively) were also retained. The UST_dominant_ had a negative relationship with swimming velocity (i.e., less time performing the in-water phase of the stroke cycle led to a faster velocity), whereas a larger F_mean_dominant_ led to a faster velocity. Imbalances in the swimming velocity achieved by each upper limb were observed at these ages and competitive levels (Morais et al., 2020a). The motion performed by the dominant limb allowed the swimmers to reach faster velocities, taking less time to perform the in-water phase of the stroke cycle (UST) and producing more propulsion (Morais et al., 2020a). Moreover, it has been shown that when under task constraint, as the SF increases, the UST becomes shorter, leading to the production of more propulsion and thus speeding up (Cohen et al., 2018). It was shown that even during all-out bouts (i.e., maximum velocity), both upper limbs have different partial contributions to swimming velocity (Morais et al., 2020a). That said, the dominant upper limb plays a key role in the swimming velocity achieved.

Besides H and F_mean_dominant_, H^2^ and F_mean_dominant_^2^ were also retained by the model. This showed that swimming velocity increased with H and F_mean_dominant_, but only up to a given extend. In this particular set of participants, the maximum velocity was achieved if H = 174.67 cm and if F_mean_dominant_ = 34.75 N. Literature clearly acknowledges the positive relationship between height and the upper limbs length for general population (Fairbanks and Fairbanks, 2005), and swimmers in particular (Kjendlie and Stallman, 2011). A study that focused on the importance of anthropometry in swimming velocity observed that the advantage of longer upper limbs could potentially be mechanically disadvantageous in some respects, as it requires muscles to apply greater force (Nevill et al., 2015). Having longer upper limbs can only be seen as an advantage if a concomitant increase in strength in the upper limbs happens. The F_mean_dominant_ peaked at 34.75 N. From this force magnitude onwards, swimming velocity decreased. Swimming velocity is characterized by a periodically accelerated motion based on the net balance between thrust (i.e., propulsion) and drag forces acting on the swimmer’s body (Barbosa et al., 2010). That is, during swimming, accelerations and deaccelerations of the swimmer’s body occur, leading to changes in velocity (known as intra-cyclic variation of the swim velocity) (Barbosa et al., 2010). Thus, swimmers can achieve faster swimming velocities when they are able to generate propulsion while reducing drag force (resistance to forward motion) (Toussaint and Beek, 1992). However, to generate higher propulsion, swimmers may suffer misalignments along their longitudinal axis which can lead to a larger frontal surface area and consequently to a higher drag (Morais et al., 2020c). Thus, despite generating higher propulsion they can be under a higher drag immediately after which will promote a decrease in their swimming velocity. Moreover, the amount of fluid that is accelerated during propulsion depends on the shape of the body and the pattern of the water flow around the body. Therefore, a heavier swimmer needs to apply a higher propulsion just to overcome inertia and dislocate added mass (Caspersen et al., 2010). This enhances the meaningful relationship between propulsion and the swimmers’ anthropometric features. Another reason can be related to the pitching and sweepback angles of the hand. Numerical studies reported that such angles have a meaningful effect on the propulsion generated by the swimmer’s hands (Bilinauskaite et al., 2013; Cohen et al., 2020). There may be specific moments of the in-water phase of the stroke cycle in which the propulsion vector is not oriented in the opposite direction of the displacement, as noted previously. In our study, swimmers were asked to perform all-out trials at maximum velocity without any constraint in their stroke mechanics. Therefore, it can be argued that greater propulsion does not always lead to faster swimming performances. This is depicted in Figure 1 (Panel E–males; Panel F–females) in which swimming velocity was analyzed against SL and considered the propulsion delivered at each SF. As such, coaches must be advised to this phenomenon to better understand that it is not “enough” to increase propulsion if swimmers do not adopt an ideal hydrodynamic profile and consequently decrease drag.

In adult/elite (Seifert et al., 2007), and youth swimming (Silva et al., 2019) it has been shown that the fastest swimmers (males and females) were characterized by a faster velocity, higher SF and longer SL compared to their slower counterparts. Moreover, the literature reports considerable insights on the practice of monitoring SF, noting the potential to determine submaximal swimming velocities above which SL will begin to drop (Barden and Kell, 2009; Koga et al., 2020). However, scarce information can be found on the combinations between the two main variables of stroke mechanics (i.e., SF and SL) that are responsible for swimming velocity in both adult/elite and young swimmers. A study by Craig and Pendergast (1979) observed an “optimal” SF–SL combination in adult/elite sprint swimmers, suggesting this to be adopted in competition. For young swimmers, our data revealed a significant effect of SL and SF for both sexes when adopting velocity as the dependent variable (SL and SF were rounded as the independent or predictor variables). Studies have reported that increases in swimming velocity in both sexes are associated with faster SF (Seifert et al., 2007; Morris et al., 2016). The improvement of SF can happen in a relatively short period of time within a training program (Girold et al., 2006). Bio-feedback training programs have been reported to be conducive to such improvements (Hermann et al., 2012). On the other hand, increasing swimming velocity based on a longer SL requires a higher training period, despite promoting energy savings (Wakayoshi et al., 1993; Barbosa et al., 2008).

Overall, there are interactions between SF–SL and swimming velocity. Figure 1 suggests that swimming velocity tends to increase with greater SL and SF, even though more evidently in females. It should be noted that the fastest swimming velocity was not achieved at the fastest SF nor at the longest SL. The fastest velocity was achieved at an “optimal” SF–SL combination. Indeed, previous research has suggested that attempts should be made to determine at what velocity and to what extend SL and SF change (Dekerle, 2006). A study by Koga et al. (2020) reported the effect of exceeding the SF at maximum swimming velocity. Swimmers were instructed to perform a SF faster than the one delivered at maximum velocity. The authors observed that swimming velocity did not significantly increase when the SF exceeded the SF at maximum velocity. However, a significant decrease in SL was noted whenever a faster SF was performed (Koga et al., 2020). Notwithstanding, our data based on a categorized SL and SF as the independent or predictor variables revealed that this relationship is not always negative (i.e., whenever SF increases, the SL decreases, and vice-versa). Rather than a clear inverse relationship, a sinusoidal profile was observed between SF and SL. That is, several SF–SL combinations can be observed. It was argued that maximum swimming velocity could not be achieved during long stroke cycles (i.e., slower SF), and that each swimmer should choose the “optimal” SF to increase his/her swimming velocity (Nakashima and Ono, 2014). The same authors reported that maximum joint torque by the upper limbs was responsible for a longer SL at the same SF. This suggests that kinetics (i.e., propulsion) might play a key role in the SF–SL relationship.

In adult/elite swimmers (Tsunokawa et al., 2019) and young swimmers (Morais et al., 2020a) it has been observed a positive association between propulsion and swimming velocity. The increase in propulsion led to an increase in swimming velocity. However, when rounding SL and SF by propulsion, a significant effect was noted in both sexes for SL. It was demonstrated that specific technique instructions allowed an increase in swimming velocity and SL with an increase in propulsion (Havriluk, 2009). However, a non-significant effect was verified on SF and on the SL-SF interaction. Indeed, the fastest swimming velocity was not achieved by the highest propulsion when rounding by SL–SF. The literature about propulsion in swimming reports that the in-water force produced by the swimmer is not always in the direction of the body’s center of mass displacement (Bilinauskaite et al., 2013; Soh and Sanders, 2021). In this case, the increase in the magnitude of the propulsion does not produce an increase in swimming velocity (Havriluk, 2009). On the other hand, it has been shown that faster SF promoted increases in propulsion and, consequently, in swimming velocity (Cohen et al., 2018). The latter study conducted a numerical simulation based on a scan of a female swimmer. The upper limbs’ motion was the same in all strokes and optimized conditions (e.g., hands’ orientation). Even though numerical simulations provide insightful information, they do not allow us to understand how different constraints can impose significant variability in motor behavior. The SF exceeding the SF at maximum velocity was shown to reduce the propulsion of the hand during the push phase, caused by the decrease in the angle of attack (Koga et al., 2020). If sprinters are instructed to perform at a very fast SF, this can result in rushing the catch phase and producing less force or a poorly space-oriented vector force. Moreover, faster SF’s are promoted by an increase in hand velocity, which could be related to more propulsion by the hand but also a reduction in the propulsion duration (i.e., impulse). It is also influenced by the ability of the swimmer to generate propulsion. If the swimmer is not “strong” enough, he/she can change the movement pattern to find less resistance in the hand, and consequently reducing the propulsion. Thus, the effective propulsion (force in the direction of the displacement) is diminished.

Overall, swimming velocity prediction retained a significant sex effect, and was determined by anthropometric, kinematic, and kinetic variables. Even though, H and F_mean_dominant_ presented a positive and significant effect, it was shown that swimming velocity started to decrease after reaching a given H and F_mean_dominant_. Coaches should be aware that longer leverages can only be an advantage if swimmers are able to produce an amount of strength that can be transferred to water (i.e., propulsion). Moreover, generating higher propulsion may not present the desired effect (i.e., fastest swimming velocity). If swimmers do not maintain a streamlined position by avoiding longitudinal misalignments, this may increase their frontal surface area and consequently drag. It was shown that maximum swimming velocity was not achieved at the highest SF or longest SL. Rather, it was achieved by an “optimal” SF–SL combination. The fastest velocity was not achieved at the highest propulsion. This may be related to the pitching and sweepback angles of the hand, which during the entire in-water phase of the stroke cycle may not be properly oriented in the opposite direction of the swimmer’s displacement. Thus, despite exerting higher amount of propulsion, it may not be mechanically advantageous if not well oriented. One must be aware that an increase in propulsion by itself may not directly lead to an increase in swimming velocity. As such, age-group coaches, and swimmers, rather than focusing exclusively on increasing SF, should find the “optimal” SF–SL combination. They must also pay attention to the swimmer’s hand orientation.

As main limitations, it can be considered that: 1) the SF–SL combinations reported in this study are only representative of the stroke mechanics of age-group sprinters without breathing actions; 2) the restrictive number of swimmers and stroke cycles included in the study, that may affect the results in an objective of generalization to a race or training performed in a 50 m swimming pool; 3) only the propulsion of the upper limbs was measured (nonetheless it accounts 90% of the total swimming velocity), and; 4) other variables than force may testify from the kinetics of the swimming motion, for instance impulse. One can suggest that future studies: 1) assess the role of propulsion in the SF–SL combination in different age-groups; 2) assess the role of propulsion in the SF–SL combination at different race paces or incremental tests, and; 3) include the kinematics of the hand to have insight on the amount of propulsion oriented in the direction of the displacement. Hand kinematics will allow us to obtain information about the amount of effective propulsion and swimming efficiency.

## Conclusion

It can be concluded that swimming velocity was predicted by an interplay of variables related to anthropometry, kinematics, and kinetics. Swimming velocity increases up to a given H and F_mean_dominant_. Upon that, anthropometric features can only play a positive and significant role on swimming velocity if swimmer’s also increase in muscle strength. Higher propulsion also plays a key-role on swimming velocity but if well orientated. Moreover, in age-group sprinters of both sexes, the fastest swimming velocity was not achieved with the fastest SF nor with the shortest SL. The fastest velocity was achieved by an “optimal” SF–SL combination. Likewise, the fastest velocity was not reached while delivering the highest propulsion. Thus, coaches must be aware that an increase by the SF may not promote a velocity increase. Same rational for the propulsion. A substantial focus must be put in the SF–SL combinations, to understand which one delivers better performance in an individual way.

## Data Availability

The original contributions presented in the study are included in the article/Supplementary Material, further inquiries can be directed to the corresponding author.
